# Identification of BACE1 cleavage sites in human voltage-gated sodium channel beta 2 subunit

**DOI:** 10.1186/1750-1326-5-61

**Published:** 2010-12-23

**Authors:** Manuel T Gersbacher, Doo Yeon Kim, Raja Bhattacharyya, Dora M Kovacs

**Affiliations:** 1 Neurobiology of Disease Laboratory, Genetics and Aging Research Unit, MassGeneral Institute for Neurodegenerative Disease, Massachusetts General Hospital, Harvard Medical School, Charlestown, MA 02129, USA

## Abstract

**Background:**

The voltage-gated sodium channel β2 subunit (Navβ2) is a physiological substrate of BACE1 (β-site APP cleaving enzyme) and γ-secretase, two proteolytic enzymes central to Alzheimer's disease pathogenesis. Previously, we have found that the processing of Navβ2 by BACE1 and γ-secretase regulates sodium channel metabolism in neuronal cells. In the current study we identified the BACE1 cleavage sites in human Navβ2.

**Results:**

We found a major (147-148 L↓M, where ↓ indicates the cleavage site) and a minor (144145 L↓Q) BACE1 cleavage site in the extracellular domain of human Navβ2 using a cell-free BACE1 cleavage assay followed by mass spectrometry. Next, we introduced two different double mutations into the identified major BACE1 cleavage site in human Navβ2: 147LM/VI and 147LM/AA. Both mutations dramatically decreased the cleavage of human Navβ2 by endogenous BACE1 in cell-free BACE1 cleavage assays. Neither of the two mutations affected subcellular localization of Navβ2 as confirmed by confocal fluorescence microscopy and subcellular fractionation of cholesterol-rich domains. Finally, wildtype and mutated Navβ2 were expressed along BACE1 in B104 rat neuroblastoma cells. In spite of α-secretase still actively cleaving the mutant proteins, Navβ2 cleavage products decreased by ~50% in cells expressing Navβ2 (147LM/VI) and ~75% in cells expressing Navβ2 (147LM/AA) as compared to cells expressing wildtype Navβ2.

**Conclusion:**

We identified a major (147-148 L↓M) and a minor (144-145 L↓Q) BACE1 cleavage site in human Navβ2. Our *in vitro *and cell-based results clearly show that the 147-148 L↓M is the major BACE1 cleavage site in human Navβ2. These findings expand our understanding of the role of BACE1 in voltage-gated sodium channel metabolism.

## Background

BACE1/β-secretase is an aspartic protease highly expressed in neuronal cells [1,2]. Together with presenilin/γ-secretase, BACE1 cleaves the amyloid precursor protein (APP) to generate amyloid β peptides (Aβ). Aβ accumulates in the brains of Alzheimer's disease patients where it promotes disease pathology [3,4]. In addition to contributing to Aβ generation, BACE1 regulates emotional memory, synaptic function and myelination in mouse brains possibly by cleaving multiple neuronal substrates [5-7]. More than 60 BACE1 substrates have recently been identified via quantitative proteomics [8]. However, only a few substrates have been investigated and confirmed *in vivo *[6,9-12]. Cleavage of substrate proteins may contribute to the important function of BACE1 in development and maintenance of the nervous system but the detailed molecular mechanism is not known.

Voltage-gated sodium channels (Nav) are composed of central α subunits and one or two accessory β subunits [13]. The pore forming α subunits regulate sodium ion transport in neuronal membranes and are therefore essential for neuronal membrane excitability [13]. The β subunits are type I transmembrane proteins with extracellular immunoglobulin and short intracellular C-terminal domains. Interaction of β subunits with α subunits regulates Nav assembly and activity [13-15]. In particular, the β2 subunit (Navβ2) regulates cell-surface expression and inactivation kinetics of Nav channels in neurons [16,17]. In addition, β subunits modulate cell adhesion and neurite outgrowth [18-20].

Previously, we and another group found that ADAM10, BACE1, and γ-secretase cleave Navβ2 in neuronal cells and mouse brains [11,12]. In a follow-up study, we showed that elevated BACE1 activity increased release of Navβ2-ICD (intracellular domain) through cleavage of Navβ2 resulting in elevated protein and mRNA levels of Nav1.1 α subunits in neuroblastoma cells [21,22]. Furthermore, processing of endogenous Navβ2 and Nav1.1 protein levels were elevated in BACE1-transgenic mouse brains and eventually resulted in altered sodium current densities in hippocampal neurons. These data strongly suggest that BACE1 can regulate neuronal function, possibly by cleaving Navβ2 in physiological conditions. In order to better understand the role of BACE1 in Nav metabolism, we have identified the BACE1 cleavage site in human Navβ2 in the present study.

## Materials and methods

### Plasmids, transfection, and reagents

Expression constructs encoding full-length human Navβ2 (GenBank: NM_004588) containing a C-terminal V5-His tag and full-length human BACE1 (GenBank: AF190725) containing a C-terminal myc tag have been described previously [11]. Navβ2 (147LM/VI) and Navβ2 (147LM/AA) were constructed using QuickChange Site-directed Mutagenesis kit (Stratagene) with the following primers: Navβ_2 _(147LM/VI): 5'-GGCAAGATCCATCTGCAGGTCGTCATTGAAGAGCCCCCTGAGCGG-3' 5'-CCGCTCAGGGGGCTCTTCAATGACGACCTGCAGATGGATCTTGCC-3'; Navβ2 (147LM/AA): 5'-GGCAAGATCCATCTGCAGGTCGCCGCGGAAGAGCCCCCTGAGCGG-3' and 5-'CCGCTCAGGGGGCTCTTCCGCGGCGACCTGCAGATGGATCTTGCC-3'. Effectene (Qiagen) was routinely used for transfecting cell lines. GL189 (Calbiochem) was used in 10 μM concentration.

### *In vitro *cleavage assay of Navβ2 substrate peptide

Navβ2 substrate peptide (β2-peptide) with N-terminal biotin was synthesized by CHI Scientific (M.W. 4049.7, purity = 94.02% determined by HPLC). A biotinylated tyrosine group was added to the N-terminus of the β2-peptide. Reaction mixtures containing 20 mg of β2-peptide, 0.1 M Na-Acetate (pH 4.0), and 2.5 mg human BACE1 (R&D systems), were prepared and incubated at 37°C for 16 h. Reactions were stopped by heating to 95°C with LDS-SDS-PAGE sample loading buffer (Invitrogen) for 5 min.

Reaction samples were then resolved on 12% BisTris gels (Invitrogen), transferred to PDVF membrane for Western blot analysis or fixed directly for silver staining. Vector ABC kit (Vector Labs) was used to detect full-length and N-terminal fragment of β2-peptide in Western Blot while Silver SNAP II kit (Invitrogen) was used to detect total protein in the gel.

### Mass spectrometry

Reaction samples from the β2-peptide *in vitro *cleavage assay were analyzed by MS using a QStarR Pulsar I (Applied Biosystems) equipped with a nanospray source (in collaboration with Proteomic core at Harvard Partners Center for Genetics and Genomics). Analyst software (Invitrogen) was used to determine the molecular weights of all cleavage products in the reaction mixture.

### Western blot analysis

Cell lysates were prepared by directly extracting cells in a buffer containing 10 mM Tris-HCl (pH 6.8), 1 mM EDTA, 150 mM NaCl, 0.25% Nonidet P-40, 1% Triton X-100, and a protease inhibitor cocktail (Roche) followed by a centrifugation at 16,000 *g*. 20-50 mg of protein were resolved on 12% BisTris gels (Invitrogen). The blots were visualized by enhanced chemiluminescence (ECL). Images were captured using BioMax film (Kodak) or VersaDoc imaging system (Biorad) and quantified with QuantityOne software (Biorad). The followings are antibodies used in this study: anti-V5 (1:5000; Invitrogen), anti-myc (1:2000: Cell Signalling) anti-GAPDH (1:2000; BD Biosciences), and antiflotillin-1 (1:250; BD Biosciences).

### *In vitro *generation of Navβ2-CTFβ

Membrane preparation and *in vitro *generation of Navβ2-CTFβ were performed as described earlier [23]. In brief, cells were washed with PBS, scraped in 1 ml PBS and centrifuged for min at 8000 rpm. Cell pellets were resuspended in 700 μl buffer H (20 mM HEPES, 150 mM NaCl, 10% glycerol, 5 mM EDTA, pH 7.4) and the solution drawn 20 times through a 3 ml syringe with 20 gauge needle. Unbroken cells were removed by centrifugation at 4000 rpm for 5 min. In order to obtain P2 fractions, the supernatant was centrifuged at 55000 rpm for 1 h and 4°C. Membrane fractions were washed once in 300 μl incubation buffer (0.1 M Na Acetate pH 4.0, 10 μg/ml Leupeptin, 1 μg/ml Aprotinin, 1 mM PNT and 5 mM EDTA) and resuspended in 100 μl incubation buffer in absence or presence of GL189. After incubation for 3 h at either 0°C or 37°C, the samples were loaded on 12% BisTris and investigated by Western blot analysis.

### Lipid Raft Fractionation

Cells were grown to 80 - 90% confluency in three 150-mm dishes, washed twice in phosphate buffered saline and scraped into 1.2 ml extraction buffer containing 0.5% Lubrol WX (Lubrol 17A17; Serva), protease inhibitor cocktail (Roche) and 1 mM phenylmethylsulfonyl fluoride (PMSF). Cells were then homogenized by five passages through a 25-gauge needle. Cell lysates were adjusted to 45% final concentration of sucrose (final volume, 4 ml) and loaded to the bottom of a 12-ml SW41 ultracentrifuge tube. A discontinuous sucrose gradient was established by sequentially layering 35% sucrose (4 ml) and 5% sucrose (4 ml) on top of the sample. Tubes were subjected to ultracentrifugation at 39,000 rpm for 18 h in Beckman SW41 rotor at 4°C. Twelve 1 ml fractions were collected from the top of the gradient and equal volume of each fraction was analyzed by Western blotting. V5 antibody was used to detect Navβ2 and flotillin-1 antibody was used as a lipid raft marker.

### Immunocytochemistry

Cells were grown on coverslips to 20 - 40% confluency and fixed with 4% paraformaldehyde for 20 min at room temperature. Cells were then rinsed three times with PBS and blocked for 1 h in PBS containing 1% BSA (Sigma-Aldrich) 0.1% Triton X-100 (FisherBiotech), 0.1% Gelatin (Sigma-Aldrich) and 0.05% Tween 20 (MP Biomedicals). Cells were incubated with anti V5 antibody for 1 h, washed three times with PBS and incubated for 30 min with rabbit anti-mouse Alexa Fluor 488 antibody (1:200: Serotec) at room temperature. After washing three times with PBS containing 0.1% Triton X-100 and 0.05% Tween-20 for 3 min the coverslips were mounted onto glass slides using Prolong Gold antifade reagent with DAPI (Invitrogen) and analyzed with a Olympus florescence microscope equipped with a confocal disk scanning unit.

## Results

### Identification of BACE1 cleavage sites in human Navβ2

**Figure 1:**
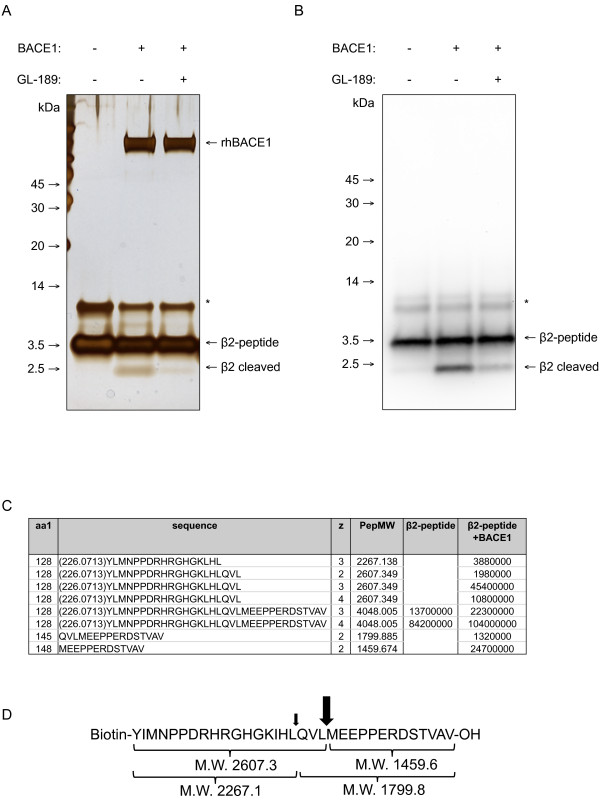
**Identification of BACE1 cleavage sites in human Navβ2 by MS analysis**. Reaction mixtures from an *in vitro *BACE1 cleavage assay were resolved on a 12% BisTris gel and full-length peptide and cleavage products were detected by silver staining. Only the incubation of the β2-peptide with BACE1 generates a ~2 kDa cleavage product. GL-189, a BACE1 inhibitor, significantly decreased the amount of cleavage product (lane 3). B) Western blot analysis of an *in vitro *BACE1 cleavage assay samples using anti-biotin-HRP conjugate. C) Table containing the major cleavage products and their predicted sequences determined by Mass spectrometry from control and the BACE1 reaction sample. D) Sequence of the β2-peptide synthesized for the *in vitro *cleavage assay. A biotinylated tyrosine amino acid is added to the N-terminus of the peptide for detection by anti-biotin-HRP conjugate. One major (big arrow) and one minor (small arrow) BACE1 cleavage site were detected by MS analysis.

To identify BACE1 cleavage sites in human Navβ2, we synthesized a substrate peptide (β2-peptide) corresponding to a 32 amino acid sequence at the juxtamembrane region of human Navβ2 (Figure 1D). A biotinylated tyrosine group was added to the N-terminus of the β2-peptide to detect N-terminal fragments. Reaction mixtures containing the β2peptide and purified recombinant human BACE1 were incubated at 37°C for 16 h. The incubation of the β2-peptide with BACE1 specifically increased cleavage products ~2 kDa, detected by both silver staining and anti-biotin-HRP conjugate (Figure 1A and 1B, lane #2). The generation of the 2 kDa cleavage products was dramatically inhibited by the BACE1 inhibitor GL-189 (Figure 1A and 1B, lane #3). The reaction samples were then analyzed by mass spectrometry (MS) to characterize all cleavage products (Figure 1C). Thus we were able to identify a major (147-148 L↓M, where ↓ indicates the cleavage site) and a minor (144-145 L↓Q) BACE1 cleavage site in the extracellular domain of human Navβ2 (Figure 1D). Interestingly, we found that the major cleavage site of human Navβ2 is slightly different from the one previously reported for mouse Navβ2 (Figure five, [12]). This difference might be due to two amino acid changes, 143(H/Y) and 148(M/L) close to the known BACE1 cleavage sites in human and mouse Navβ2.

### Mutations of the BACE1 cleavage site decrease BACE1-mediated processing of human Navβ2 in purified CHO cells membrane

**Figure 2:**
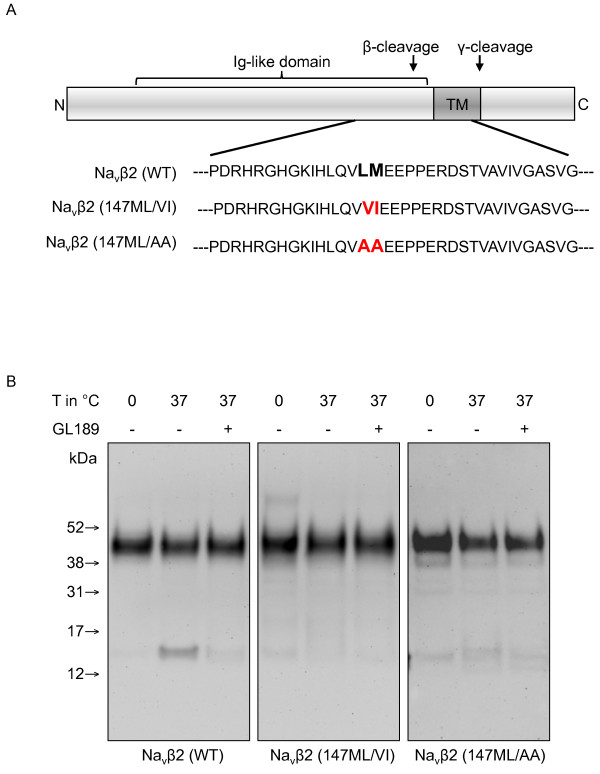
**Characterization of the major BACE1 cleavage site in an *in vitro *assay**. A) Schematic representation of the Navβ2 (147ML/VI) and Navβ2 (147ML/AA) mutations introduced into the identified major BACE1 cleavage site. B) Cell-free generation of Navβ2-CTF in membranes from CHO cells expressing Navβ2 wildtype, Navβ2 (147ML/VI) or Navβ2 (147ML/AA). Treatment with the BACE inhibitor GL189 inhibited cleavage of wildtype Navβ2. Navβ2-CTF generation is nearly abolished in Navβ2 (147ML/VI) and Navβ2 (147ML/AA).

To specifically measure BACE1-mediated cleavage of Navβ2, we used a cell-free BACE1-cleavage assay [21]. We introduced two separate double mutations, 147LM/VI and 147LM/AA at the S1 and S1' positions of the major BACE1 cleavage site (Figure 2A). Wildtype and mutated human Navβ2 were expressed in CHO cells and crude membrane fractions prepared from those cells were incubated for 3 h at pH 4.5 with or without the BACE1 inhibitor GL-189. As expected, wildtype Navβ2 (WT) was cleaved at 37°C to generate β2-CTFβ, which is completely blocked by treatment with GL-189 (Figure 2B, left panel). However, the cleavage of Navβ2 (147LM/VI) and Navβ2 (147LM/AA) is dramatically decreased (Figure 2B, middle and right panel). These data strongly indicate that 147-148 L↓M is the major BACE1 cleavage site in human Navβ2.

### Mutations of the BACE1 cleavage site do not affect subcellular localization of human Navβ2

**Figure 3:**
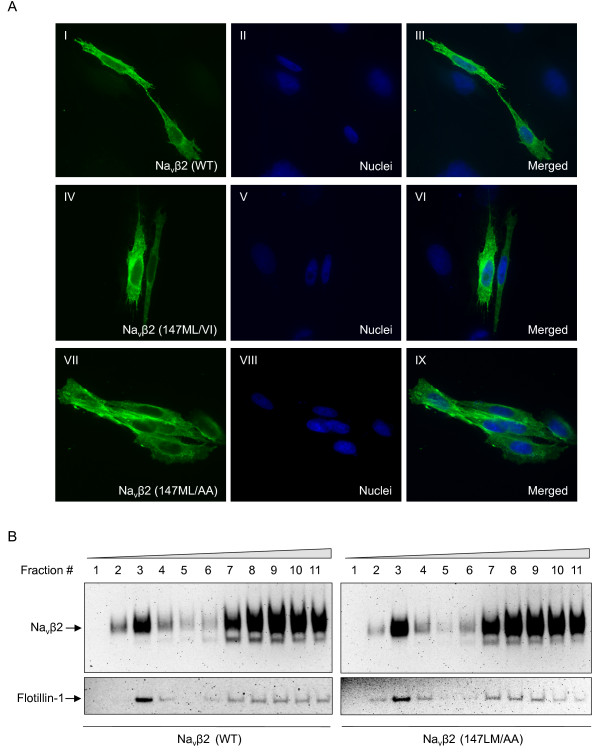
**Similar subcellular localizations of Navβ2 (147LM/VI) and Navβ2 (147LM/AA) as compared to wildtype Navβ2**. A) Confocal images of enriched cultures expressing Navβ2 wildtype, Navβ2 (147ML/VI) or Navβ2 (147ML/AA) immunostained with a C-terminal V5 antibody. B) Western blot analysis of cells expressing either wildtype Navβ2 or Navβ2 (147LM/AA) subjected to sucrose gradient fractionation. Flotillin-1 staining marks lipid raft fractions. The Navβ2 (147ML/VI) and Navβ2 (147ML/AA) mutations in the BACE1 cleavage site did not visibly affect subcellular or lipid raft localization of Navβ2.

To exclude the possibility that the mutations introduced into Navβ2 caused its retention in early compartments, we studied the subcellular localization of the mutated versus wildtype Navβ2. We established enriched cultures of CHO cells transfected with wildtype and mutated Navβ2 and investigated localization of Navβ2 by florescence confocal microscopy. We could not detect any significant difference in the subcellular localization of mutated Navβ2 as compared to wildtype Navβ2 (Figure 3A). These data were also confirmed in B104 cells (data not shown).

Next, we tested whether the distribution of Navβ2 into cholesterol-rich domains (lipid rafts) is altered by the 147LM/AA mutation. As previously reported, Navβ2 was detected in lipid raft enriched fractions, confirmed by flotillin-1 staining (Figure 3B, fractions 2 to 4, [12]). We could not detect any significant changes in the levels of Navβ2 (147LM/AA) in lipid-raft fractions. Together, these results suggest that localization of Navβ2 was not affected by mutations of the BACE1 cleavage site.

### Mutations of the BACE1 cleavage site decrease processing in cell based models

**Figure 4:**
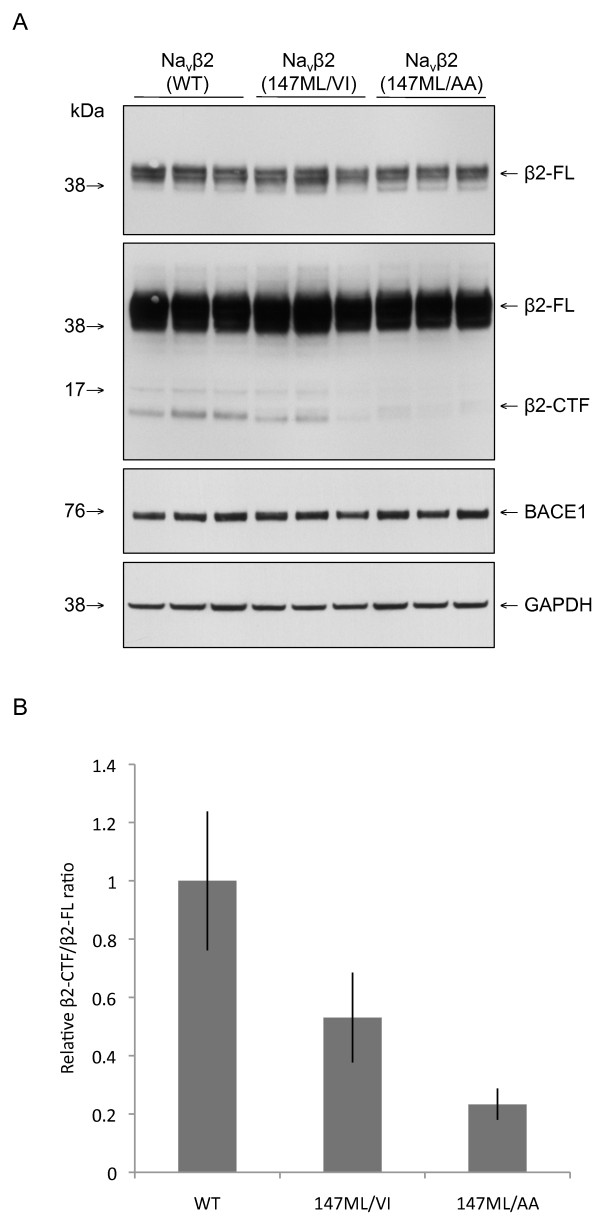
**Mutations in the BACE1 cleavage site strongly decrease processing of Navβ2 in B104 cells overexpressing BACE1**. A) B104 cells stably overexpressing BACE1 were transfected with Navβ2 wild-type, Navβ2 (147ML/VI) or Navβ2 (147ML/AA) and then subjected to Western blot analysis. B) Quantification of Navβ2-CTF to Navβ2-FL ratios from A. Navβ2-CTF levels were decreased by mutations in the BACE1 cleavage site (FL, full length; CTF, C-terminal fragment).

To further investigate the identified major BACE1 cleavage site (147-148 L↓M), we tested whether 147LM/VI and 147LM/AA mutations would also reduce Navβ2 cleavage in cells. Wildtype and mutated Navβ2 were transfected into B104 rat neuroblastoma cells stably overexpressing BACE1. α-secretase-mediated processing of Navβ2 would not be affected by mutations in the BACE1 cleavage site. Therefore, we did not expect complete inhibition of Navβ2 processing in cells expressing the mutated form of the protein. Indeed, we found that Navβ2-CTF production was decreased by ~50% in cells expressing Navβ2 (147LM/VI) and reduced by ~75% in cells expressing Navβ2 (147LM/AA) as compared to wildtype Navβ2 expressing cells (Figure 4A and 4B). In addition, we transfected wildtype Navβ2 and Navβ2 (147LM/VI) together with BACE1 into CHO cells. Similar to B104 cells, mutation of the identified BACE cleavage site strongly reduced Navβ2-CTF production (data not shown). These data confirm that 147-148 L ↓ M is the major BACE1 cleavage site in human Navβ2.

## Discussion

**Figure 5:**
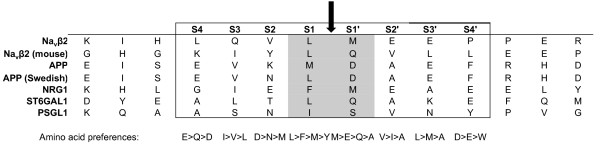
**Cleavage sequence comparison among BACE1 substrates**. A black arrow indicates the currently characterized BACE1 cleavage sites of BACE1 substrates. Amino acid preferences around the BACE1 cleavage sites were determined by Turner R.T. et al. [24]. Interestingly, human Navβ2 harbors the preferable residues at S1 and S1', the most important positions for BACE1 recognition and cleavage.

Using a similar synthetic peptide substrate and MALDI-MS, Wong *et al*. previously reported that 144-145 L↓Q is the major BACE1 cleavage site of mouse Navβ2 [12]. It is interesting that the major cleavage site of human Navβ2 is slightly different from that of mouse Navβ2 (Figure 1D and 5). The mouse BACE1 cleavage site of Navβ2 is similar to the minor (144-145 L↓Q) BACE1 cleavage site in the human Navβ2 identified in our study. This site difference may be due to amino acid differences between human and mouse Navβ2. As shown in Figure 5 there are two amino acid changes between human and mouse, in proximity to the BACE1 cleavage sites Navβ2 (143(H/Y) and 148(M/L)). Particularly, the 148(M/L) site is located in the S1' position of the BACE1 cleavage site of human Navβ2. According to Turner et al., a methionine at S1' position in human Navβ2 is highly preferable for BACE1 cleavage as compared to a leucine [24]. Further studies will be required to confirm this cleavage site in brains and to explain whether changes in the BACE1 cleavage site between species would lead to a differential regulation of sodium channel metabolism in human as compared to mouse.

A recent study identified 68 BACE1 substrates, underscoring the role of BACE1 in various cellular processes [8]. Similarly to Navβ2, the majority of those substrates are, type 1 transmembrane proteins with extracellular N-terminal and intracellular C-terminal ends. However, in addition to Navβ2 only a few BACE1 substrates have been confirmed under physiological conditions. These include APP, Neuregulin 1/3 (NRG-1/3), alpha 2,6-sialyltransferase (ST6GAL1), and P-selectin glycoprotein ligand-1 (PSGL1) [6, 912]. As shown in Figure 5 the BACE1 cleavage sites in these substrates show highly similar sequences. For example, the S1 cleavage position has been suggested as the most important site for determining BACE1-mediated processing based on an *in vitro *cleavage assay using synthetic substrate peptides [24]. The majority of substrates harbor leucine, phenylalanine, or methionine at the S1 cleavage site. Interestingly, human Navβ2 harbors both leucine and methionine at the S1 and S1' position, which are the most preferable residues at those positions according to *in vitro *studies [24]. These studies also suggest, that subsites proximal to the scissile bond (S1 and S'1) are more stringent than distal residues which is reflected by the drastic decrease in CTF production in Navβ2 harboring mutations 147LM/AA or 147LM/VI.

In our previous studies, we have found that elevated BACE1-mediated cleavage of human Navβ2 increased mRNA and protein levels of Nav1.1 α subunit by increasing the release of the β2 intracellular domain (Navβ2-ICD) [21]. Therefore, it will be interesting to see whether the blockage of BACE1 cleavage in Navβ2 147LM/IV and Navβ2 147 LM/AA would also decrease Nav1.1 levels by decreasing Navβ2-CTF levels and possibly Navβ2-ICD levels. However, these studies are difficult because α-secretase cleavage of Navβ2 is not affected by the 147 LM/VI and 147 LM/AA mutations. On the contrary, we have observed elevated α-secretase cleavage in CHO cells transiently expressing Navβ2 (147 (LM/AA) (data not shown). The α-secretase cleavage site is distinct from and closer to the membrane than the BACE1 cleavage site, unlikely to be directly affected by the BACE1 cleavage site mutations [21,25]. It is more likely that the elevated α-secretase cleavage observed only in CHO cells is due to a compensatory αsecretase-mediated cleavage since BACE1 and α-secretase seem to compete for juxtamembrane cleavages as shown in APP processing. Additional mutations completely blocking α-secretase-mediated cleavages will be required to fully address the role of BACE1 in sodium channel metabolism in normal conditions.

BACE1 levels and activities are significantly elevated in AD brains, possibly contributing to the disease progression [26-28]. BACE1 levels are also increased in some injury conditions including brain trauma [29] and ischemia [30-32], suggesting a possible role of BACE1 as a stress-response protein [2]. The fact that BACE1 regulates Nav channel metabolism via Navβ2 suggests the interesting possibility that BACE1 might modulate sodium channel metabolism not only in AD but also in other disease conditions in which BACE1 levels are increased. It will be interesting to test whether the BACE1cleavage mutation in Navβ2 reported here would also alter sodium channel metabolism in various stress conditions including oxidative stress and mitochondrial dysfunctions, which are known to increase BACE1 levels.

## Conclusion

We identified a major (147-148 L↓M) and a minor (144-145 L↓Q) BASE1 cleavage site in human Navβ2 by using a synthetic β2-peptide and MS. We also found that mutations of the major BACE1 cleavage site (147LM/VI and 147 LM/AA) dramatically decreased BACE1-mediated cleavage of human Navβ2 in an *in vitro *assay and a cell based model. Our data clearly demonstrate that the BACE1 cleavage site (147-148 L↓M) is mainly responsible for BACE1 cleavage of human Navβ2.

## Abbreviations used

APP: amyloid precursor protein; Aβ: amyloid β peptide; Nav: voltage-gated sodium channel; MS: mass spectrometry; Navβ2: voltage-gated sodium channel β2 subunit; BACE1: β-site APP cleaving enzyme; β2-peptide: Navβ2 substrate peptide; CHO: Chinese hamster ovary; CTF: C-terminal fragment; FL: full-length; ICD: intracellular domain

## Competing interests

The authors declare that they have no competing interests.

## Authors' contributions

MTG and DYK participated in the design of the study and drafted the manuscript. MTG carried out the *in vitro *cleavage assay, immunofluorescence imaging and Western blotting. DYK did the *in vitro *cleavage assay and MS. RB carried out the lipid raft fractionation and helped to draft the manuscript. DMK conceived of the study, participated in its design and coordination and helped to draft the manuscript. All authors read and approved the final manuscript.
